# A rapid near-patient detection system for SARS-CoV-2 using saliva

**DOI:** 10.1038/s41598-021-92677-z

**Published:** 2021-06-28

**Authors:** Noah B. Toppings, Abu Naser Mohon, Yoonjung Lee, Hitendra Kumar, Daniel Lee, Ratik Kapoor, Gurmukh Singh, Lisa Oberding, Omar Abdullah, Keekyoung Kim, Byron M. Berenger, Dylan R. Pillai

**Affiliations:** 1 Department of Microbiology, Immunology, and Infectious Diseases, University of Calgary, Calgary, AB Canada; 2 Department of Mechanical and Manufacturing Engineering, University of Calgary, Calgary, AB Canada; 3 School of Engineering, University of British Columbia, Kelowna, BC Canada; 4 Biomedical Engineering Graduate Program, University of Calgary, Calgary, AB Canada; 5 Clinical Section of Microbiology, Alberta Precision Laboratories, Calgary, AB Canada; 6 Department Pathology and Laboratory Medicine, University of Calgary, Calgary, AB Canada; 7 Clinical Section of Infectious Diseases, Department of Medicine, University of Calgary, Calgary, AB Canada

**Keywords:** Biotechnology, Assay systems, Infectious diseases, Viral infection

## Abstract

The highly infectious nature of SARS-CoV-2 necessitates the use of widespread testing to control the spread of the virus. Presently, the standard molecular testing method (reverse transcriptase-polymerase chain reaction, RT-PCR) is restricted to the laboratory, time-consuming, and costly. This increases the turnaround time for getting test results. This study sought to develop a rapid, near-patient saliva-based test for COVID-19 (Saliva-Dry LAMP) with similar accuracy to that of standard RT-PCR tests. A lyophilized dual-target reverse transcription-loop-mediated isothermal amplification (RT-LAMP) test with fluorometric detection by the naked eye was developed. The assay relies on dry reagents that are room temperature stable. A device containing a centrifuge, heat block, and blue LED light system was manufactured to reduce the cost of performing the assay. This test has a limit of detection of 1 copy/µL and achieved a positive percent agreement of 100% [95% CI 88.43% to 100.0%] and a negative percent agreement of 96.7% [95% CI 82.78–99.92%] relative to a reference standard test. Saliva-Dry LAMP can be completed in 105 min. Precision, cross-reactivity, and interfering substances analysis met international regulatory standards. The combination of ease of sample collection, dry reagents, visual detection, low capital equipment cost, and excellent analytical sensitivity make Saliva-Dry LAMP particularly useful for resource-limited settings.

## Introduction

Due to the highly infectious nature of SARS-CoV-2 and its ability to be transmitted by asymptomatic individuals^1^, widespread testing for COVID-19 is critically important to preventing the spread of the virus^1^. The COVID-19 pandemic has put immense demands on molecular testing infrastructure^2,3^. Presently, the standard method for COVID-19 diagnostic testing is reverse-transcriptase polymerase chain reaction (RT-PCR)^4–6^. This method cannot be deployed outside of a laboratory. However, for the sake of contact tracing and self-isolation, the utility of a test relates to how quickly one can receive the results of the test after the sample is obtained^7,8^. Tests which require transporting samples to a centralized laboratory increases this time. Not surprisingly, governments have pushed for the immediate development of rapid, near-patient tests for COVID-19^9,10^. Near-patient tests must yield straight-forward results which are easy to interpret. For remote settings, these tests should not be reliant on cold-chains and sophisticated equipment. To meet this immediate need, we developed Saliva-Dry LAMP, a rapid, near-patient saliva test for COVID-19 that uses lyophilized dual-target reverse transcriptase-loop-mediated isothermal amplification (RT-LAMP) with fluorometric detection by the naked-eye^11^. This test can be performed on a portable and low-cost device that we manufactured.

## Methods

### Patient samples and ethics

Clinical specimens used in this study were anonymized saliva from individuals in Alberta collected between May and September 2020. No clinical information was obtained. Saliva or nasopharyngeal (NP) swabs were collected in universal transport media (UTM) (COPAN Diagnostics Inc., Murrieta, USA) for ease of use^12^. The research involves human participants and was performed in accordance with relevant guidelines/regulations. Informed consent was obtained from all participants, and was approved by Conjoint Health Research Ethics Board (CHREB) at the University of Calgary (REB20-0402/0444).

### RNA extraction

The RNA extraction method was tested with undiluted saliva (neat saliva), however, this resulted in the spin columns getting clogged. Instead of adding additional steps to overcome clogging, saliva was collected in UTM. Saliva diluted in UTM (approximately 25% saliva, 75% UTM, 140 µL total) was mixed with 560 µL of a concentrated preparation of lysis buffer and spiked with 2 µL of 50,000 pfu/µL MS2 bacteriophage (Zeptometrix, Buffalo, NY). Buffers used are described previously by Zainabadi et al*.*^13^*.* This lysate was hand shaken, then incubated at 61 °C for 5 min. The lysate was applied to a spin column (Omega Bio-Tek Inc., Norcross, USA) and spun in a mySPIN™ 12 (Thermo Fisher Scientific Inc., Waltham, USA) for 110 s at a peak speed of 11,300 RPM. The flow-through was discarded and 500 µL of wash 1 was applied to the column. The column was centrifuged again (110 s, 11,300 RPM) and the flow-through discarded. Next, 500 µL of wash 2 was applied to the column before centrifugation for 170 s at a peak speed of 11,300 RPM. Columns were then transferred to new collection tubes and 50 µL of elution buffer was added. RNA was eluted with a final spin (110 s, 11,300 RPM).

### Lyophilized RT-LAMP Reactions (“Dry LAMP”)

**Figure 1:**
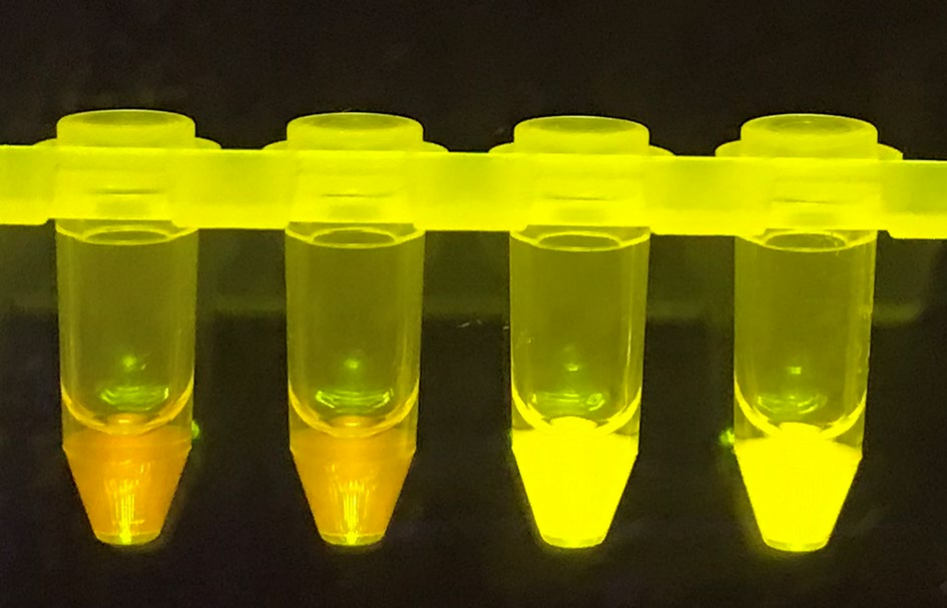
Visual results of Saliva-Dry LAMP reactions as viewed under a blue light transilluminator. The two reactions on the left are negative (orange) and the two reactions on the right (bright green) are positive read outs.

Lyophilized RT-LAMP reactions for the detection of SARS-CoV-2 were prepared by Pro-Lab Diagnostics Inc. (Richmond Hill, Canada) using patented dual-target primers^14^ and the GspSSD2.0 Isothermal Mastermix (ISO-004) (OptiGene Ltd., Horsham, UK). These pellets were dissolved in 10 µL resuspension buffer R1 (Pro-Lab Diagnostics Inc.) and 0.5 µL of dye mix (5.95 mM hydroxynapthanol blue trisodium salt, 69.5 X GelGreen^Ⓡ^) (Biotium, Fremont, USA). Next, 14.5 µL of extracted RNA was added to each dissolved pellet. Reactions were mixed and then 30 µL of mineral oil was added on top. Reactions were incubated for 45 min at 61 °C in an IncuBlock™ Mini Dry Bath (Thomas Scientific, Swedesboro, USA) then visualized under an LED transilluminator (MaestroGen Inc., Hsinchu City, ROC). Positive reactions appeared bright green while negative reactions appeared orange (Fig. 1). Reactions intermediate between orange and bright green were called positive in order to maximize sensitivity. When a viewer could not decide how to call a reaction, another individual called the reaction on their own.

MS2 external amplification controls were ran in parallel in separate lyophilized RT-LAMP reactions prepared by Pro-Lab Diagnostics Inc. using the primers from Benzine et al*.*^15^. Lyophilized reactions were dissolved with 15 µL of resuspension buffer R1, 0.5 µL dye mix and 4.5 µL elution buffer. Reactions contained 5 µL of extracted RNA. Reactions were run simultaneously with SARS-CoV-2 reactions at 61 °C for 45 min and visualized as described above. These lyophilized MS2 reactions were used with both the Biobox and commercially-available instruments.

### Saliva reference RT-PCR

Two different reference standard RT-PCR tests were used to evaluate the performance of Saliva-Dry LAMP. In the first reference RT-PCR, RNA extracted from saliva samples using the streamlined column-based extraction which was used for Saliva-Dry LAMP was also used in RT-PCR reactions. This assessed the performance of the lyophilized RT-LAMP chemistry relative to RT-PCR. For this first RT-PCR test, the US Centres for Disease Control and Prevention N2-gene assay^17^ as well as the Alberta Precision Labs E-gene assay^16^ were performed. For the E-gene assay, 5 µL of extracted RNA was added to 2.5 µL of TaqMan Fast Virus One-Step RT-PCR Master Mix, 0.4 µL of each forward and reverse primers (800 nM final concentration), 0.2 µL of probe (200 nM final concentration), and 1.5 µL of nuclease-free water. Reactions were run on a CFX-96 (Bio-Rad Laboratories Inc., Hercules, USA) with the following thermocycling parameters: 50 °C for 5 min, 95 °C for 20 s, and then 45 cycles of 95 °C for 3 s and 60 °C for 30 s. Cycle threshold (Ct) values were determined using the Bio-Rad proprietary non-linear regression algorithm. The CDC N2 assay was ran in the same manner as below, except it was run on a CFX-96 instrument.

### Nasopharyngeal swab reference RT-PCR method

In the second reference RT-PCR, saliva and NP swabs were collected concomitantly to assess the performance of the entire Saliva-Dry LAMP workflow relative to an entire FDA Emergency Use Authorization test^17^. This second reference RT-PCR test was the US Centres for Disease Control and Prevention N1/N2/RNase P RT-PCR test performed according to CDC-006-00019, Revision: 01^17^. For this test, the QIAGEN QIAamp viral RNA mini kit was used to extract 140 µL of NP swab UTM which was finally eluted in 50 µL of AE buffer^18^. For RT-PCR, 5 µL of extracted RNA was added to 5 µL of TaqPath™ 1-Step RT-qPCR Master Mix, 1.5 µL of combined primer/probe mix, and 8.5 µL of nuclease-free water^17^. Reactions were run on an Applied Biosystems™ 7500 Fast Dx Real-Time PCR Instrument using the following thermocycling profile: 25 °C for 2 min, 50 °C for 15 min, 95 °C for 2 min, and then 45 cycles of 95 °C for 3 s, 55 °C for 30 s^17^. Entirely different sample sets were used for each of the two comparator tests (saliva versus saliva and saliva versus NP swab).

### Droplet digital-PCR

A high titre positive sample was quantified using a Bio-Rad QX200™ Droplet Digital™ (dd) PCR system (Bio-Rad Laboratories, Hercules, CA) using the same E-gene RT-PCR primers following extraction with the SV Total RNA Isolation System (Promega Corp., Madison, USA)^19^. The ddPCR master mix consisted of (per sample) 2.5 μL One-Step RT–ddPCR reverse transcriptase, 6.25 μL One-Step RT–ddPCR Supermix, 1 μL 300 mmol/L dithiothreitol, 1 μL of each forward and reverse primers, 0.5 μL probe (20 μM primers and 10 μM probe), 7.5 µL RNase-free water, and 5 μL of extracted RNA. A 20 µL aliquot of each template mastermix was added to the sample well of the droplet generation cartridge, with 70 μL of droplet generation oil for probes. Thermocycling was done with the Bio-Rad C1000 Touch™ Thermal Cycler before measurement with the QX200™. Cycling conditions were 50 °C for 1 h, 95 °C for 10 min, 40 cycles of 95 °C for 30 s and 60 °C for 60 s, then 98 °C for 10 min. Ramp rates were 2 °C/s.

### Limit of detection studies

The limit of detection using commercially-available instruments and the Biobox was determined using a patient sample (NP swab diluted in 25% saliva, 75% UTM) which was quantified by ddPCR. This sample was serially diluted to achieve a range from 1 to 0.25 copies/μL. The saliva:UTM ratio of 1:3 was chosen based on two factors. Firstly, standard UTM collection tubes contain 3 mL of UTM. Secondly, the average volume of saliva collected was observed to be approximately 1 mL (data not shown) and this is supported by the results of Lagerlöf and Dawes^20^.

### Cross-reactivity and interfering substance studies

Potentially cross-reactive respiratory pathogens were tested with Saliva-Dry LAMP using inactivated stocks from Zeptometrix (Buffalo, USA) (Table S2). For interference testing (Table S3), SARS-CoV-2 negative samples and samples contrived to 9X LOD (9 copies/µL) or 3X LOD (3 copies/µL) were spiked at the indicated concentrations with substances expected to be commonly found in saliva. For interference testing, samples were contrived using a high titre positive NP swab (the same one used for the limit of detection studies) in 25% saliva and 75% UTM + interfering substance (the volume of interfering substance added was subtracted from the volume of UTM which would have been added to achieve 75% UTM).

### Clinical validations

Saliva is not collected routinely for COVID-19 diagnosis in Alberta. Given the low prevalence of COVID-19 in Alberta during this study, saliva and corresponding NP swab samples had to be collected from individuals who previously tested positive by RT-PCR. Clinical saliva samples were selected to reflect the natural distribution of viral loads in the population during early infection (See Figures S1 and S2). Plots, and 95% confidence intervals (Clopper-Pearson) were made using MATLAB R2020b (The Mathworks Inc., Natick, USA).

### Biobox fabrication

**Figure 2:**
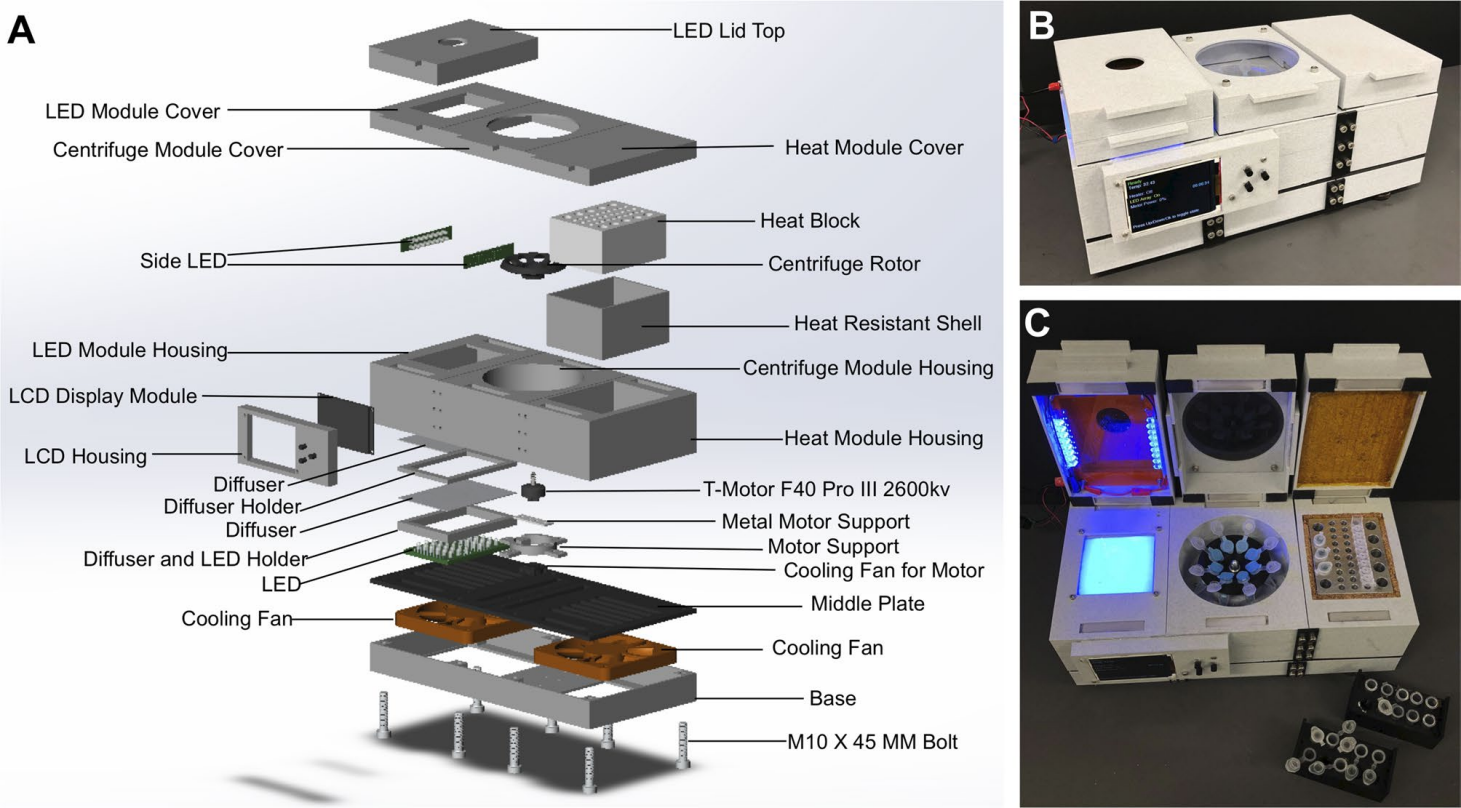
The Biobox was Biobox manufactured to perform the Saliva-Dry LAMP experiments. (**A**) Computer-aided design drawing with exploded view of the Biobox. The device is comprised of a heat block, centrifuge, and blue LED transilluminator which met specifications to perform the Saliva-Dry LAMP reaction. Photographs of the side (**B**) and top (**C**) view of the Biobox are shown for reference**.**

A custom-made device, termed “Biobox” (Fig. 2), was developed to execute the sequence of steps for Saliva-Dry LAMP—centrifugation, isothermal incubation and naked-eye fluorescent detection respectively. The Biobox comprised of three components—centrifuge, heating block and transilluminator (470 nm light emitting diode, LED, arrays). The design was prepared using Solidworks™ 2020 (Dassault Systems, Waltham, USA). All housing parts/fixtures were fabricated using a fused deposition modeling (FDM) 3D printer (Anycubic C, Commerce, USA) with poly-lactic acid (PLA) filament unless specified. The centrifuge rotor was fabricated using polycarbonate filament. The transilluminator consists of two LED arrays—a 6 × 8 LED array mounted inside the Biobox and a pair of 2 × 8 LED arrays mounted on the sides of the cap to provide illumination from the sides. A second cap was placed on the transilluminator with acrylic sheet window to block the wavelengths emitted by the LED’s but not the intercalating dye. The aluminum heating block was machined to house both 2 mL and 1.5 mL microcentrifuge tubes. The temperature of the heating block was maintained at 61 °C using three heating elements and three thermocouple sensors. The centrifuge was made with a direct current (DC) powered brushless motor (T-motor F40 Pro3 2600 kV, Nanchang, P.R.C.) mounted on an aluminum bracket. The centrifuge rotor was mounted on the brushless motor and achieved 8000 RCF. All components were controlled by an ESP32 microprocessor. The device is operated through the user interface using an LCD display and pushbuttons. A DC power supply of 21–23 V was used to power the device.

### Saliva-dry LAMP performed on the biobox

The lyophilized RT-LAMP reagents for the amplification of SARS-CoV-2 on the Biobox were obtained from Illucidx Inc. (Calgary, Canada). Lyophilized reactions consisted of the master mix described previously^14^ but employed the dye mix described above. A proprietary excipient mix was also added. For extractions on the Biobox, conditions were identical as those on the commercially-available instruments with the exception that centrifugation times were 50 s shorter (due to faster ramping). Lyophilized reactions were resuspended with 25 µL of extracted RNA, mixed, then 30 µL of mineral oil was added on top. LAMP was run for 45 min at 61 °C and visualized with the Biobox LED transilluminator.

## Results

### Analytical study of Saliva-Dry LAMP

**Figure 3:**
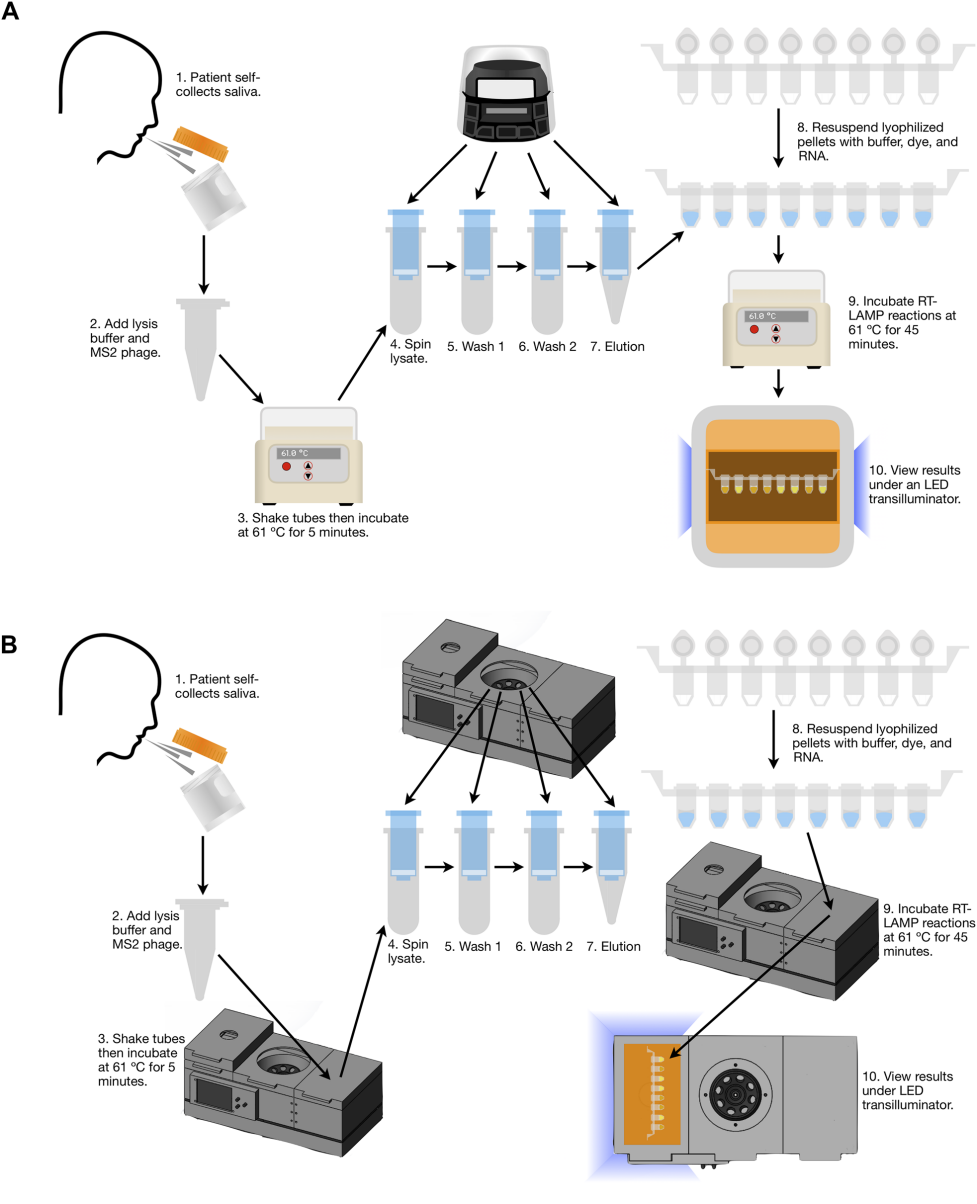
Saliva-Dry LAMP workflow diagram and equipment requirements. The workflow for conducting Saliva-Dry LAMP on (**A**) commercially-available instruments and (**B**) the Biobox.

The Saliva-Dry LAMP workflow, on the Biobox, is depicted in Fig. 3. The limit of detection was determined with commercially-available instruments and the Biobox using a dilution series of a quantified contrived sample which spanned 1–0.25 copies/µL (Table 1). All replicates tested positive at 1.0 copies/µL when using commercially-available instruments and the Biobox with their respective LAMP chemistry combinations (Table 1). A limit of detection confirmation was conducted on commercially-available instruments using 20 replicates at 0.5 copies/μL prepared in the same way as described previously. This confirmation failed as only 14 of 20 replicates were positive (data not shown). The limit of detection confirmation was then retried successfully on commercially-available instruments at 1 copy/μL (19/20 positive) (data not shown).

**Table 1:** Limit of detection determined by dilution series for Saliva-Dry LAMP using commercially-available instruments and the Biobox manufactured in this study.

Sample concentration (copies/μL)	Positive reactions	
	Commercially-available instruments	Biobox
1.0	4/4	4/4
0.5	4/4	3/4
0.25	0/4	2/4

A single experiment performed in quadruplicate is shown at each concentration using a contrived saliva sample containing SARS-CoV-2.

### Clinical Validation compared to nasopharyngeal swab RT-PCR

This clinical validation was conducted on 60 unique clinical saliva samples (~ 25% saliva, ~ 75% UTM) using commercially-available instruments and lyophilized RT-LAMP reactions from Pro-Lab Diagnostics Inc. Considering that no gold standard method existed for saliva at the time of experimentation, positive percent agreement (PPA) and negative percent agreement (NPA) was calculated (Table 2). The CDC reference RT-PCR was run on the corresponding NP swabs as reference method (Fig. S1). Saliva-Dry LAMP achieved a PPA of 100% (30/30) [95% CI 88.43% to 100.0%] and an NPA of 96.7% (29/30) [95% CI 82.78–99.92%] (Table 3).

**Table 2:** Saliva-Dry LAMP clinical validation using paired saliva and NP swabs obtained compared to the reference standard CDC RT-PCR method.

Saliva-Dry LAMP (S + RdRP)	CDC RT-PCR on paired NP swab (N1 and N2 gene)		Total
	Positive	Negative	
Positive	30	1	31
Negative	0	29	29
Total	30	30	60
PPA	100% [95% CI 88.43–100.0%]		
NPA	96.7% [95% CI 82.78–99.92%]		

Saliva-Dry LAMP was performed using commercially-available instruments and lyophilized RT-LAMP reactions from Pro-Lab Diagnostics Inc.

*PPA* positive percent agreement, *NPA* negative percent agreement, *S* spike, *RdRP* RNA-dependent RNA polymerase.

**Table 3:** Saliva-dry-LAMP clinical samples compared to reference RT-PCR methods (E gene and N2 gene) for saliva.

Saliva-Dry LAMP (S + RdRP)	RT-PCR on Saliva RNA extracts (E and N2 gene)		Total
	Positive	Negative	
Positive	28	0	28
Negative	2	33	35
Total	30	33	63
PPA	93.33% (95% CI 77.93–99.18%)		
NPA	100.00% (95% CI 89.42–100.00%)		

Saliva-dry-LAMP was performed using commercially-available instruments and lyophilized RT-LAMP reactions from Pro-Lab Diagnostics Inc.

*PPA* positive percent agreement, *NPA* negative percent agreement, *S* spike, *RdRP* RNA-dependent RNA polymerase.

### Clinical Validation compared to Saliva RT-PCR

This clinical validation was conducted on 63 unique clinical saliva samples (~ 25% saliva, ~ 75% UTM) using commercially available intruments and lyophilized RT-LAMP reactions from Pro-LAB Diagnostics Inc. positive percent agreement (PPA) and negative percent agreement (NPA) were calculated (Table 3). CDC N2-gene and E-gene RT-PCR were run on the replicates as a reference method (Figure S2). Saliva-Dry LAMP achieved a PPA of 93.33% (28/30) [95% CI 77.93–99.19%] and an NPA of 100.00% (33/33) [95% CI 89.42–100.00%] (Table 3). Two samples were indicated as positive by both RT-PCR’s and negative by Saliva-Dry LAMP. One sample was barely positive by E-gene RT-PCR and negative by N2-gene RT-PCR which was considered negative.

### Assay precision, cross-reactivity, and interference

These experiments were done using commercially-available instruments and lyophilized RT-LAMP reactions from Pro-Lab Diagnostics Inc.. None of the 11 potentially cross-reactive respiratory pathogens tested showed any cross reactivity with Saliva-Dry LAMP in vitro (Table S1). None of the 18 potentially interfering medicines/substances tested showed any interference with Saliva-Dry LAMP in vitro at 9X LOD (9 copies/µL)(Table S2). However, at 3X LOD (3 copies/µL), a positive result was unexpectedly not obtained with the addition of human blood, Robitussin Mucus and Phlegm, and Chloraseptic® sore throat spray (Table S2). The assay precision was confirmed with two samples twice a day for 20 days (Table S3). Variation arising from equipment differences was determined to be adequate with five samples per day on three different sets of instruments for five days (Table S4).

### Capital cost of saliva-dry LAMP

When using a set of commercially-available instruments, the capital cost of the instruments required for the test is US $1977.44 with over half of that cost coming from the mySPIN™ 12 mini centrifuge (Table S5). The cost of all of the major components of the Biobox is much lower at US $386.72 (Table S6).

## Discussion

In this study, we have developed a rapid, near-patient, saliva test for COVID-19 using lyophilized LAMP reagents with fluorometric detection by the naked-eye. Experiments were designed to satisfy regulatory standards^21^. Saliva-Dry LAMP demonstrated excellent positive and negative agreement with RT-PCR performed on both concomitantly collected NP swabs and the saliva RNA extracts used for Saliva-Dry LAMP (Tables 2 and 3). The lowest agreement in either of the clinical validations was a positive percent agreement of 93.33% (95% CI 77.93% to 99.18%) for the saliva RNA extracts (Table 3). The one false positive (when comparing Saliva-Dry LAMP to the FDA Emergency Use Authorization reference RT-PCR test) was likely a sampling error for the NP swab as the concomitant saliva sample was positive (CDC N2 RT-PCR positive, Ct 25.97). Reports of a patient’s saliva but not NP swab testing positive for SARS-CoV-2 are widespread^12,22,23^. Saliva-Dry LAMP showed no cross-reactivity from any of the respiratory pathogens tested (Table S1). Interference was observed with 3 of 18 potentially interfering substances tested at 3 X LOD (3 copies/µL), but not at 9 X LOD (9 copies/µL) (Table S2). Saliva collection instructions can be amended to exclude samples containing noticeable blood, Chloraseptic® sore throat spray, and Robitussin Mucus and Phlegm. In silico analysis by Mohon et al*.* did not identify any primer cross-reactivity in 13 relevant respiratory pathogens^14^. However, the clinical validation set did not contain many samples with very late Ct values in the 35–40 range which in our experience fail to amplify by LAMP. Due to the paucity of in vivo studies, the clinical and epidemiological significance of high Ct value (> 35), low viral copy, individuals remains unclear^24,25^. In vitro studies suggest individuals with low viral loads are rarely infectious or not infectious^24,25^. The analytical sensitivity of a diagnostic test is not the only measure by which it should be judged^8^. In terms of limiting the transmission of SARS-CoV-2, the advantages of Saliva-Dry LAMP are its superior sample-to-result time (~ 105 min) compared to RT-PCR, near-patient deployment, ease of application, and cost^8^.

The performance characteristics, limit of detection, and ease-of-use of Saliva-Dry LAMP are comparable to some near-patient nucleic acid COVID-19 tests. STOPCovid is a rapid diagnostic test that employs LAMP and CRISPR technology but relies on NP swabs^26^. Saliva-Dry LAMP achieved similar sensitivity as STOPCovid (93.1%, 188/202) and specificity (98.5%, 197/200)^26^. Lalli et al*.* developed an extraction-free RT-LAMP test for COVID-19 using saliva with colorimetric detection^27^. Lalli et al*.* achieved a slightly higher limit of detection than Saliva-Dry LAMP (59 copies/reaction or 21.6 copies/µL of saliva) but the clinical validation achieved a sensitivity of 85% (17/20) and a specificity of 90% (9/10)^27^. A limitation of this study is that the Saliva-Dry LAMP limit of detection experiments were conducted with a quantified high titre NP swab sample (which was diluted in 25% saliva, 75% UTM) instead of diluting a high titre saliva sample. Further limit of detection experiments should be done starting with a quantified saliva sample to eliminate any artifacts caused by the NP swab.

As an RT-LAMP test, Saliva-Dry LAMP uses different reagents than RT-PCR, thus averting some supply chain bottlenecks and export restrictions^2,3,28^. Saliva-Dry LAMP uses saliva instead of the standard NP swab, as saliva is more amenable to rapid, point-of-care testing in resource-limited settings, while still having an acceptable sensitivity compared to that achieved with NP swabs^12,22^. Saliva can be collected without a healthcare worker^22,29^ and self-collection does not induce coughing, sneezing or bleeding^29,30^. Therefore, saliva collection avoids depleting critical supplies of PPE and swabs while reducing healthcare worker demand and exposure^22,29^. Saliva is also favourable for testing children as NP swabs are invasive^29^.

An important area of ongoing development for point-of-care nucleic acid tests is rapid RNA extraction. Standard laboratory RNA extractions are very time-consuming; however, replacing an RNA purification step with a simple inactivation step can compromise assay sensitivity^14,31,32^. RNA purifications result in the concentration of viral RNA and the removal of amplification inhibitors, both of which increase sensitivity. Some rapid RNA extraction methods exist, but many of them require a cold-chain^26,27,33,34^. The streamlined, column-based RNA extraction developed for Saliva-Dry LAMP purifies RNA in under 30 min while concentrating RNA 2.8-fold and costing only US $2.46 per preparation.

Simpler workflows exist for processing saliva samples for nucleic acid amplification tests than the method presented here. One example is the COVID-19 RT-LAMP test developed by Yamazaki et al*.* which digests saliva with a semi-alkaline protease for 15 min followed by inactivation at 95 °C for 5 min^35^. The SalivaDirect COVID-19 test uses a similar processing method although it utilizes RT-PCR and is therefore not a point-of-care test^33^. For SalivaDirect, neat saliva is mixed directly with proteinase K at which point the sample is vortexed vigorously, transferred to another tube, and then inactivated by heating to 95 °C for 5 min^33^.

Using commercially-available equipment, this test has a throughput of 10 samples per batch. Manufacture of the Biobox instrument reduced the capital equipment cost fivefold from US $1977.44 to US $386.72 (Table S5 and S6), while still achieving an excellent limit of detection. The Biobox instrument has a throughput of seven patient samples per batch when using an extraction negative control. Saliva-Dry LAMP has a capital equipment cost an order of magnitude less than RT-PCR when using commercially-available instruments, and a capital equipment cost nearly two orders of magnitude less than RT-PCR when using the Biobox (S. Rudgar, personal communication). Either of these options enables the deployment of Saliva-Dry LAMP in resource-limited settings.

The Illucidx lyophilized reactions ran on the Biobox performed at a similar level as the Pro-Lab Diagnostics lyophilized reactions on commercially-available instruments despite the Illucidx lyophilized reactions using 2.9-fold more extracted RNA. The benefit of using Illucidx RT-LAMP reactions is that they do not require the external addition of dye and resuspension buffer requiring refrigeration. With regards to the MS2 external amplification controls, these reactions have a limited ability to serve as controls for RT-LAMP inhibitors in the SARS-CoV-2 reactions as they use a smaller volume of extracted RNA than the SARS-CoV-2 reactions. Any inhibitors present would be more diluted in the MS2 reactions, attenuating any inhibitory effects. However, using less RNA for the MS2 controls gives the user the flexibility to re-run RT-LAMP on RNA extracts without having to re-extract the sample if a mistake is made during reaction setup.

There are various ‘all in one’ machines for genetic analysis outside of a laboratory. Bento Lab is a mobile genetics setup capable of performing PCR and running and visualizing electrophoresis gels which bears similarities to the Biobox presented here^36^. However, the Bento Lab has different intended uses and so it is not suitable for Saliva-Dry LAMP. Specifically, unlike the Biobox, the Bento Lab cannot perform column-based nucleic acid extractions due to the performance limitations of its centrifuge (our observations and ref.^36^). Unlike the Biobox, the Bento Lab can only utilize one of its elements at a time^36^. For these reasons, an entirely different device needed to be developed to perform Saliva-Dry LAMP. When compared to major commercially-available point of care tests however, the limitations of the Biobox are more apparent.

The Lucira™ COVID-19 All-In-One Test Kit is an at home point-of-care test for COVID-19 which uses self-collected nasal swab samples^37^. The Lucira™ test utilizes lyophilized RT-LAMP reactions to detect SARS-CoV-2 RNA using a battery-powered handheld device^37^. In a clinical validation, the Lucira™ test achieved a PPA of 94.1% (95% CI 85.5–98.4%) and an NPA of 98.0% (95% CI 89.4–99.9%) relative to a high sensitivity FDA authorized SARS-CoV-2 assay^37^. An RNA purification step is not used in the Lucira™ test and results are displayed automatically as soon as possible^37^. Notwithstanding kit availability in different jurisdictions, the Lucira™ test is superior to Saliva-Dry LAMP in most aspects except for the lower throughput of the Lucira™ test (one sample per kit)^37^.

Another limitation of this study is that the two clinical validations were performed on commercially-available instruments, not on the Biobox. Thus, the performance of the Biobox with clinical samples is unknown. However, given that the Biobox and commercially-available instruments achieved the same limit of detection (Table 1), it is inferred that the performance of the Biobox with clinical samples would be highly similar to the performance with commercially-available instruments. Future experiments should test clinical samples on the Biobox.

A limitation of all tests which rely on visually identifying a color change—including Saliva-Dry LAMP—is that there is some subjectivity involved in calling reactions positive or negative based on their color (especially when a reaction resembles both the positive and negative colors). However, with the fluorescent dye mix used here, positive and negative reactions differed from one another in both color and emitted light intensity, making the contrast between positive and negative reactions two-fold (unlike tests which are merely colorimetric)^38–41^. In the future, a mobile phone app which discriminates specific colors could be used to call reactions, thus reducing subjectivity^39^.

Saliva-Dry LAMP has its limitations. Firstly, the positive controls, dye, and resuspension buffer used in this study are not room-temperature stable reagents and require a cold chain. Second, while the sample-to-result time near the patient is useful, the time required to perform the test is approximately 105 min with a minimal throughput of four samples per run. In particular, the silica spin column-based RNA extraction has many pipetting steps which increases both the required time, and the risk of introducing laboratory contamination. Finally, the equipment developed here still requires electricity and further refinements to increase portability. A second prototype of the Biobox relying on a lithium-ion battery is feasible and is currently being evaluated. Future studies will aim to port Saliva-Dry LAMP onto a microfluidic cartridge, improving speed, and point-of-care feasibility. Presently, the value of Saliva-Dry LAMP for resource-limited settings lies in its synergy of dry reagents, visual detection, ease of sample collection, excellent analytical sensitivity, and low capital cost.

## Supplementary Information

Supplementary Information.

## Data Availability

The datasets generated during and/or analysed during the current study are available from the corresponding author on reasonable request.
