# Supplementary material for: A rapid near-patient detection system for SARS-CoV-2 using saliva
Source: Sci Rep. 2021 Jun 28;11:13378. doi: 10.1038/s41598-021-92677-z (PMC8238998; doi:10.1038/s41598-021-92677-z)
Supplement: Supplementary file 1 — Supplementary Information. [file 41598_2021_92677_MOESM1_ESM.docx]

**A rapid near-patient detection system for SARS-CoV-2 using saliva**

**Supplementary Materials:**

Table S1. Saliva-LAMP demonstrates no cross-reactivity with a panel of 11 respiratory pathogens

| **Pathogen** | **Strain** | **Concentration** | **Result** | **Agreement with Expected Results** |
| --- | --- | --- | --- | --- |
| ***Staphylococcus aureus*** | **COL (MRSA)** | 10-fold dilution of stock that yields a Ct value of 18.5-21 by PCR | Negative | 100% (1/1) |
| ***Streptococcus pneumonia*** | **Z022** | 10-fold dilution of stock that yields a Ct value of 26-28 by PCR | Negative | 100% (1/1) |
| **Influenza A H3** | **A/Brisbane/10/07** | 10-fold dilution of stock that yields a Ct value of 22-25 by RT-PCR | Negative | 100% (1/1) |
| **Influenza B** | **B/Florida/02/06** | 10-fold dilution of stock that yields a Ct value of 19-22 by RT-PCR | Negative | 100% (1/1) |
| **RSV A2** | **N/A** | 10-fold dilution of stock that yields a Ct value of 29-32 by RT-PCR | Negative | 100% (1/1) |
| **Coronavirus 229E** | **N/A** | 10-fold dilution of stock that yields a Ct value of 25-28 by RT-PCR | Negative | 100% (1/1) |
| **Coronavirus NL63** | **N/A** | 10-fold dilution of stock that yields a Ct value of 27-30 by RT-PCR | Negative | 100% (1/1) |
| **MERS** | **Florida/USA-2_Saudi Arabia_2014** | 10-fold dilution of stock that yields a Ct value of 25-28 by RT-PCR | Negative | 100% (1/1) |
| **Coronavirus OC43** | **N/A** | 10-fold dilution of stock that yields a Ct value of 25-28 by RT-PCR | Negative | 100% (1/1) |
| **SARS** | **2003-00592** | 10-fold dilution of stock that yields a Ct value of 25-28 by RT-PCR | Negative | 100% (1/1) |
| **Coronavirus HKU1** | **N/A** | 10-fold dilution of stock that yields a Ct value of 22-25 by RT-PCR | Negative | 100% (1/1) |
| Positive Control (SARS-CoV-2, MS2) | | 10 X LOD | Positive | 100% (1/1) |
| Negative Control | | **-** | Negative | 100% (1/1) |

Table S2. Saliva-LAMP is impervious to a wide variety of household medications and substances that may be in patient samples. †Samples were prepared to 9 X LOD before they underwent an additional freeze-thaw step, further reducing the viral titre by an unknown factor. NTC: No Template Control. The 9 X LOD treatment is 9 copies/µL and the 3 X LOD treatment is 3 copies/µL.

| **Substance/Trade Name** | **Active Ingredient** | **Ingredient Concentration in Spiked Saliva-UTM Sample** | **Viral Titre in Input Saliva-UTM Sample†** | **% Agreement with Expected Results** |
| --- | --- | --- | --- | --- |
| Tylenol Extra Strength | Acetaminophen | Approximately 1.7 mg/mL | 9 X LOD | 100% (1/1 Positive) |
|  |  |  | 3 X LOD | 100% (1/1 Positive) |
|  |  |  | NTC | 100% (1/1 Negative) |
| Aspirin Extra Strength | Acetylsalicylic Acid | Approximately 12.3 mg/mL | 9 X LOD | 100% (1/1 Positive) |
|  |  |  | 3 X LOD | 100% (1/1 Positive) |
|  |  |  | NTC | 100% (1/1 Negative) |
| Colgate Total Mouthwash | Cetyopridinium Chloride | 0.005% (w/w) | 9 X LOD | 100% (1/1 Positive) |
|  |  |  | 3 X LOD | 100% (1/1 Positive) |
|  |  |  | NTC | 100% (1/1 Negative) |
| Cepacol® Sore Throat Lozenges | Benzocaine and Menthol | Approximately 0.08 mg/mL benzocaine and 0.02 mg/mL menthol | 9 X LOD | 100% (1/1 Positive) |
|  |  |  | 3 X LOD | 100% (1/1 Positive) |
|  |  |  | NTC | 100% (1/1 Negative) |
| Chloraseptic® Sore Throat Spray | Phenol | 0.07% (w/v) | 9 X LOD | 100% (1/1 Positive) |
|  |  |  | 3 X LOD | 0% (0/1 Positive) |
|  |  |  | NTC | 100% (1/1 Negative) |
| Dristan Tablets | Chlorpheniramine Maleate, Acetaminophen, Phenylephrine HCl | Approximately 0.06 mg/mL (Chloropheniramine maleate), Approximately 6.5 mg/mL (Acetaminophen), Approximately 0.1 mg/mL (Phenylephrine HCl) | 9 X LOD | 100% (1/1 Positive) |
|  |  |  | 3 X LOD | 100% (1/1 Positive) |
|  |  |  | NTC | 100% (1/1 Negative) |
| Dimetapp | Bromopheniramine Maleate, Phenylephrine HCl | 0.064 mg/mL (Bromopheniramine Maleate), 0.16 mg/mL (Phenylephrine HCl) | 9 X LOD | 100% (1/1 Positive) |
|  |  |  | 3 X LOD | 100% (1/1 Positive) |
|  |  |  | NTC | 100% (1/1 Negative) |
| Colgate Triple Action Fluoride Toothpaste | Sodium fluoride | Approximately 0.075% (w/w) | 9 X LOD | 100% (1/1 Positive) |
|  |  |  | 3 X LOD | 100% (1/1 Positive) |
|  |  |  | NTC | 100% (1/1 Negative) |
| Benadryl Extra Strength | Diphenhydramine HCl | Approximately 2.7 mg/mL | 9 X LOD | 100% (1/1 Positive) |
|  |  |  | 3 X LOD | 100% (1/1 Positive) |
|  |  |  | NTC | 100% (1/1 Negative) |
| HALLS® Kids Sore Throat Lozenges Strawberry Flavoured | Menthol | Approximately 0.08 mg/mL | 9 X LOD | 100% (1/1 Positive) |
|  |  |  | 3 X LOD | 100% (1/1 Positive) |
|  |  |  | NTC | 100% (1/1 Negative) |
| Extra Strength Ibuprofen Liquid Gel Capsules | Ibuprofen | Approximately 6 mg/mL | 9 X LOD | 100% (1/1 Positive) |
|  |  |  | 3 X LOD | 100% (1/1 Positive) |
|  |  |  | NTC | 100% (1/1 Negative) |
| Listerine Freshburst | Menthol, Thymol, and Eucalyptol | 0.0042% (w/v) menthol%, 0.0063 (w/v) thymol, 0.0091% (w/v) eucalyptol | 9 X LOD | 100% (1/1 Positive) |
|  |  |  | 3 X LOD | 100% (1/1 Positive) |
|  |  |  | NTC | 100% (1/1 Negative) |
| Rub A535 | Methyl Salicylate, Eucalyptus Essential Oil | 0.0060% (w/w) (Methyl Salicylate), 0.0006% (w/w) (Eucalyptus Essential Oil) | 9 X LOD | 100% (1/1 Positive) |
|  |  |  | 3 X LOD | 100% (1/1 Positive) |
|  |  |  | NTC | 100% (1/1 Negative) |
| Benylin Extra Strength Dry Cough | Dextromethorphan HBr | 0.2 mg/mL | 9 X LOD | 100% (1/1 Positive) |
|  |  |  | 3 X LOD | 100% (1/1 Positive) |
|  |  |  | NTC | 100% (1/1 Negative) |
| Robitussin Cough & Cold Extra Strength | Dextromethorphan HBr, Guaifenesin, Pseudoephedrine Hydrochloride | 0.2 mg/mL (Dextromethorphan Hydrobromide), 2.67 mg/mL (Guaifenesin), 0.4 mg/mL (Pseudoephedrine Hydrochloride) | 9 X LOD | 100% (1/1 Positive) |
|  |  |  | 3 X LOD | 100% (1/1 Positive) |
|  |  |  | NTC | 100% (1/1 Negative) |
| Robitussin Mucus and Phlegm | Guaifenesin | 2.0 mg/mL | 9 X LOD | 100% (1/1 Positive) |
|  |  |  | 3 X LOD | 0% (0/1 Positive) |
|  |  |  | NTC | 100% (1/1 Negative) |
| Jamieson Extra Strength Zinc Supplement (50 mg) | Zinc Gluconate | Approximately 1 mg/mL | 9 X LOD | 100% (1/1 Positive) |
|  |  |  | 3 X LOD | 100% (1/1 Positive) |
|  |  |  | NTC | 100% (1/1 Negative) |
| Human Blood | N/A | 10% (v/v) | 9 X LOD | 100% (1/1 Positive) |
|  |  |  | 3 X LOD | 0% (0/1 Positive) |
|  |  |  | NTC | 100% (1/1 Negative) |
| No Interfering Substances | N/A | N/A | 9 X LOD | 100% (1/1 Positive) |
|  |  |  | 3 X LOD | 100% (1/1 Positive) |
|  |  |  | NTC | 100% (1/1 Negative) |

Table S3. Assessment of precision of saliva-LAMP using five samples on three sets of instruments each day for five days (3 X 5 X 5).

|  | **Instrument Set 1 (mySPIN 12 HSG10802, Mini Dry Bath 061-16031-20030404, Maestrogen UltraSlim LED Translilluminator 657052301512)** | | | | | **Instrument Set 2 (mySPIN HSG10975, Mini Dry Bath 061-16031-19080030, Maestrogen UltraSlim LED Translilluminator 657028801512)** | | | | | **Instrument Set 3 (MySPIN 75004081, Mini Dry Bath 061-16031-20030359, Maestrogen UltraSlim LED Translilluminator 657017101512)** | | | | |
| --- | --- | --- | --- | --- | --- | --- | --- | --- | --- | --- | --- | --- | --- | --- | --- |
|  | **Saliva-LAMP Call** | | | | | **Saliva-LAMP Call** | | | | | **Saliva-LAMP Call** | | | | |
|  | **Day 1** | **Day 2** | **Day 3** | **Day 4** | **Day 5** | **Day 1** | **Day 2** | **Day 3** | **Day 4** | **Day 5** | **Day 1** | **Day 2** | **Day 3** | **Day 4** | **Day 5** |
| **Negative control** | Negative | Negative | Negative | Negative | Negative | Negative | Negative | Negative | Negative | Negative | Negative | Negative | Negative | Negative | Negative |
| **Positive control** | Positive | Positive | Positive | Positive | Positive | Positive | Positive | Positive | Positive | Positive | Positive | Positive | Positive | Positive | Positive |
| **Replicate 1 (10 copy/µL)** | Positive | Positive | Positive | Positive | Positive | Positive | Positive | Positive | Positive | Positive | Positive | Positive | Positive | Positive | Positive |
| **Replicate 2 (10 copy/µL)** | Positive | Positive | Positive | Positive | Positive | Positive | Positive | Positive | Positive | Positive | Positive | Positive | Positive | Positive | Positive |
| **Replicate 3 (10 copy/µL)** | Positive | Positive | Positive | Positive | Positive | Positive | Positive | Positive | Positive | Positive | Positive | Positive | Positive | Positive | Positive |
| **Replicate 4 (10 copy/µL)** | Positive | Positive | Positive | Positive | Positive | Positive | Positive | Positive | Positive | Positive | Positive | Positive | Positive | Positive | Positive |
| **Replicate 5 (10 copy/µL)** | Positive | Positive | Positive | Positive | Positive | Positive | Positive | Positive | Positive | Positive | Positive | Positive | Positive | Positive | Positive |

Table S4. Assessment of precision of saliva-LAMP using two samples twice per day for

twenty days (20 X 2 X 2).

|  | | **Negative Control** | **Positive Control** | **Replicate 1 (10 copy/µL)** | **Replicate 2 (10 copy/µL)** |
| --- | --- | --- | --- | --- | --- |
| **Day 1** | **Round 1** | Negative | Positive | Positive | Positive |
|  | **Round 2** | Negative | Positive | Positive | Positive |
| **Day 2** | **Round 1** | Negative | Positive | Positive | Positive |
|  | **Round 2** | Negative | Positive | Positive | Positive |
| **Day 3** | **Round 1** | Negative | Positive | Positive | Positive |
|  | **Round 2** | Negative | Positive | Positive | Positive |
| **Day 4** | **Round 1** | Negative | Positive | Positive | Positive |
|  | **Round 2** | Negative | Positive | Positive | Positive |
| **Day 5** | **Round 1** | Negative | Positive | Positive | Positive |
|  | **Round 2** | Negative | Positive | Positive | Positive |
| **Day 6** | **Round 1** | Negative | Positive | Positive | Positive |
|  | **Round 2** | Negative | Positive | Positive | Positive |
| **Day 7** | **Round 1** | Negative | Positive | Positive | Positive |
|  | **Round 2** | Negative | Positive | Positive | Positive |
| **Day 8** | **Round 1** | Negative | Positive | Positive | Positive |
|  | **Round 2** | Negative | Positive | Positive | Positive |
| **Day 9** | **Round 1** | Negative | Positive | Positive | Positive |
|  | **Round 2** | Negative | Positive | Positive | Positive |
| **Day 10** | **Round 1** | Negative | Positive | Positive | Positive |
|  | **Round 2** | Negative | Positive | Positive | Positive |
| **Day 11** | **Round 1** | Negative | Positive | Positive | Positive |
|  | **Round 2** | Negative | Positive | Positive | Positive |
| **Day 12** | **Round 1** | Negative | Positive | Positive | Positive |
|  | **Round 2** | Negative | Positive | Positive | Positive |
| **Day 13** | **Round 1** | Negative | Positive | Positive | Positive |
|  | **Round 2** | Negative | Positive | Positive | Positive |
| **Day 14** | **Round 1** | Negative | Positive | Positive | Positive |
|  | **Round 2** | Negative | Positive | Positive | Positive |
| **Day 15** | **Round 1** | Negative | Positive | Positive | Positive |
|  | **Round 2** | Negative | Positive | Positive | Positive |
| **Day 16** | **Round 1** | Negative | Positive | Positive | Positive |
|  | **Round 2** | Negative | Positive | Positive | Positive |
| **Day 17** | **Round 1** | Negative | Positive | Positive | Positive |
|  | **Round 2** | Negative | Positive | Positive | Positive |
| **Day 18** | **Round 1** | Negative | Positive | Positive | Positive |
|  | **Round 2** | Negative | Positive | Positive | Positive |
| **Day 19** | **Round 1** | Negative | Positive | Positive | Positive |
|  | **Round 2** | Negative | Positive | Positive | Positive |
| **Day 20** | **Round 1** | Negative | Positive | Positive | Positive |
|  | **Round 2** | Negative | Positive | Positive | Positive |

Table S5. Capital cost of Saliva-dry LAMP commercially acquired instrument.

| **Instrument** | **Cost Per Unit (CAD)** |
| --- | --- |
| mySPIN™ 12 Mini Centrifuge | $1582.20 [36] |
| IncuBlock™ Mini Dry Bath | $252.00 [37] |
| 2 X Aluminum blocks (for 0.2 mL and 2 mL tubes) | $174 [37] |
| Ultra Slim LED Transilluminator | $526.75 [38] |
| **Total Capital Cost** | **$2534.95 (US$1977.44, Dec 8, 2020 exchange rate)** |

Table S6. Capital cost of Saliva-dry LAMP Biobox (major items listed)

| **Instrument** | **Cost Per Unit (CAD)** |
| --- | --- |
| Fan | $56.19 |
| Cable | $46.38 |
| Heat block & machining | $130.29 |
| Motor | $38.43 |
| Other | $224.46 |
| **Total Capital Cost** | **$495.75 (US$386.72, Dec 8, 2020 exchange rate)** |


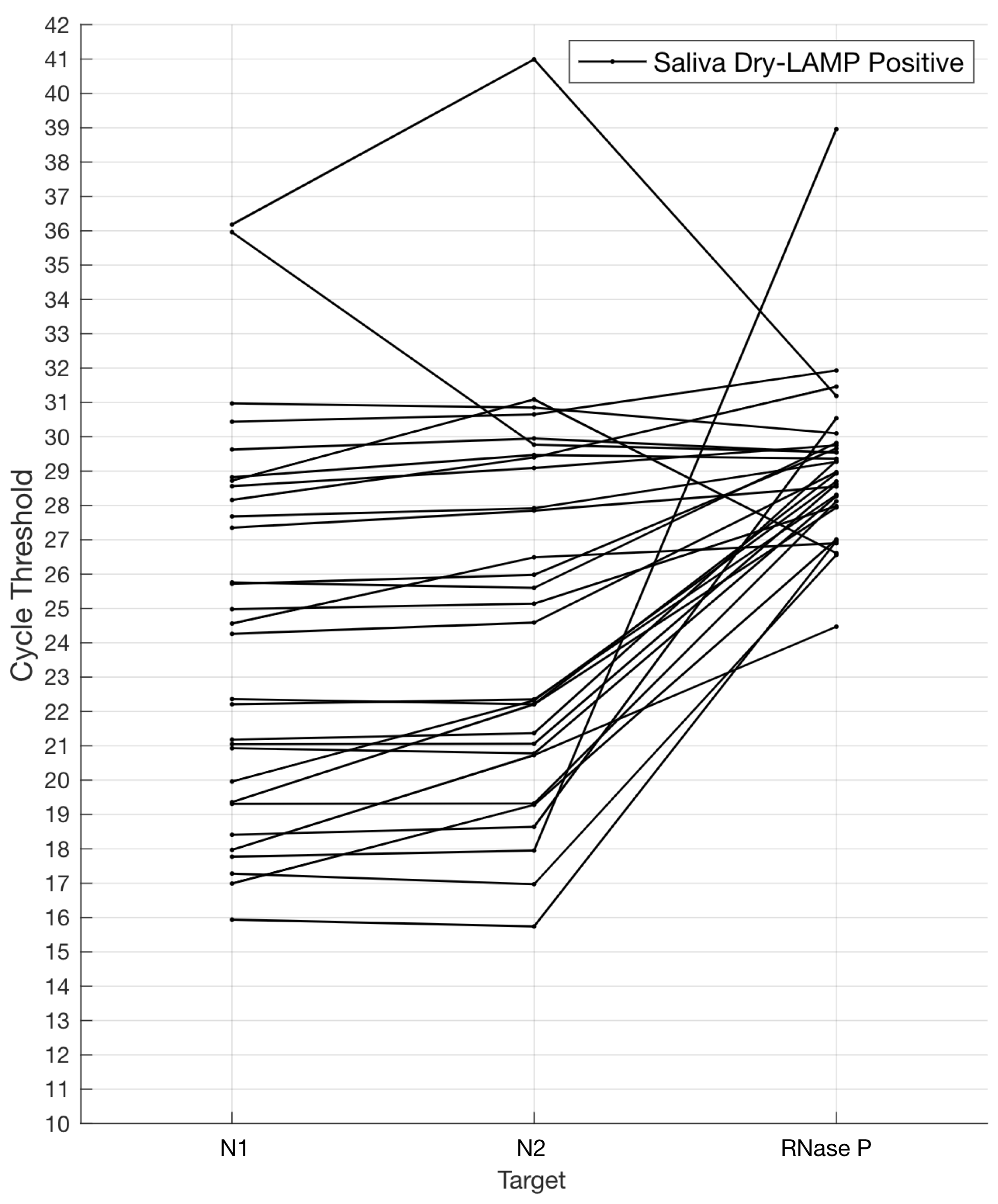


Figure S1. Cycle threshold (Ct) values from the FDA EUA reference RT-PCR for the concomitant nasopharyngeal swabs for all SARS-CoV-2-positive saliva samples used in the clinical validation (n=30).


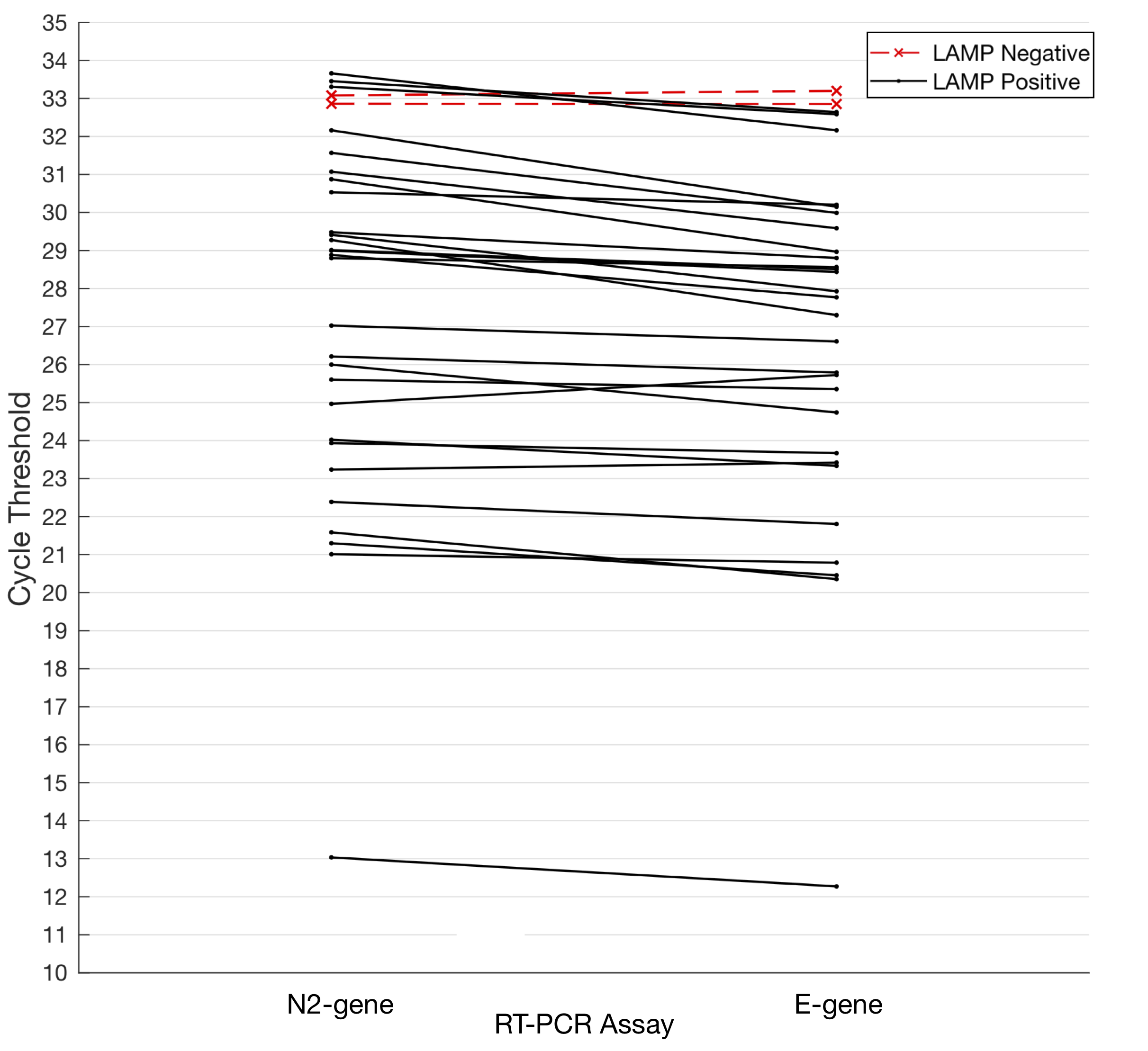
Figure S2. Cycle threshold (Ct) values for the two reference RT-PCR assays run on the RNA extracts extracted with the Saliva Dry-LAMP RNA extraction method and used for the RT-LAMP reactions. All SARS-CoV-2-positive saliva samples used in the clinical validation are shown (n=30).
